# The study of the relevance of macro- and microelements in the hair of young wrestlers depending on the style of wrestling

**DOI:** 10.3389/fendo.2022.985297

**Published:** 2022-08-11

**Authors:** Victoria Zaborova, Oxana Zolnikov, Natiya Dzhakhaya, Elena Bueverova, Alla Sedova, Anastasia Kurbatova, Victor Putilo, Maria Yakovleva, Igor Shantyr, Igor Kastyro, Mariusz Ozimek, Dmitry Korolev, Natella Krikheli, Konstantin Gurevich, Katie M. Heinrich

**Affiliations:** ^1^ Institute of Clinical Medicine, I.M. Sechenov First Moscow State Medical University, Moscow, Russia; ^2^ Moscow Institute of Physics and Technology, Dolgoprudny, Russia; ^3^ I.M. Sechenov First Moscow State Medical University, Moscow, Russia; ^4^ Nikiforov Russian Center of Emergency and Radiation Medicine, Ministry of Emergency Situations, St. Petersburg, Russia; ^5^ Peoples’ Friendship University of Russia, Moscow, Russia; ^6^ Institute of Sport, Department of Track and Field’s Sports, University of Physical Education, Krakow, Poland; ^7^ Moscow State University of Medicine and Dentistry, Moscow, Russia; ^8^ UNESCO Chair, Moscow State University of Medicine and Dentistry, Moscow, Russia; ^9^ Research Institute of Health Organization and Medical Management of the Moscow City Health Department, Moscow, Russia; ^10^ Department of Kinesiology, College of Health and Human Sciences, Kansas State University, Manhattan, KS, United States

**Keywords:** trace elements, hair, athletes, sodium, magnesium, calcium, rubidium, potassium

## Abstract

While participating in an intensive training process, the athlete’s body requires not only energy, but also specific macro- and microelements. The purpose of this study was to show the meaning of monitoring the level of mineral trace elements in athletes-wrestlers during physical activity. As an experimental group, 66 male wrestlers aged 18-20 years with at least 3 years of intensive wrestling experience were examined. The control group consisted of 92 young cadets of military school aged 18-20 years, who had previous sports training, but were not engaged in wrestling. To determine the quantitative content of trace elements, the hair was cut from the back of the head for the entire length in an amount of at least 0.1 g. an examined using the mass spectrometer ICP-MS Agilent 7900. Strong positive correlations were found for sodium with potassium and rubidium, magnesium with calcium, potassium with rubidium, and rubidium with caesium among wrestlers. Wrestlers were found to have higher levels of a number of macro- and microelements, including toxic ones.

## Introduction

When undergoing intensive physical training, an athlete’s body requires not only energy, but also a specific balance of macro- and microelements. Minerals that enter the human body with water and food are used for metabolic needs and act as cofactors of key vitamins and enzymes, among others. Due to diverse functions of minerals, young athletes often track their presence, with one such method using a hair sample (1). As well, different sport specializations have demonstrated differences in the balance of macro- and microelements. Thus, for male athletes with repetitive aerobic movements, deficiencies of calcium (Ca), cobalt (Co), copper (Cu), magnesium (Mg), phosphorus (P), zinc (Zn), iodine (I), potassium (K), and nitrogen (N) are described (2). In gymnasts, an increase in selenium (Se), Zn, and Cu has been described (3). The development of microelement imbalance reduces training process efficiency and sports performance and increases the recovery time after playing sports (4).

Differences in microelement imbalance between well-trained athletes and those with low physical activity have been established, such that the athletes had lower levels of toxic microelements including cadmium (Cd) and lead (Pb) (5). Physical training “until failure” also leads to specific changes in the balance of microelements (6). Also important is the duration of training in the development of microelement imbalance in athletes (7, 8). The role of iron (Fe) in the ability of athletes to adequately respond to the training process is actively discussed. Iron deficiency is accompanied by reduced adjustment to physical load, such as lower aerobic power (9, 10). At iron deficiency the serum ferritin level (i.e., total body iron stores) changes (11) and can result in microcytosis (12).

The role of gender in the development of iron deficiency among female athletes has been thoroughly studied in relation to the menstrual cycle (13). Women in sports are more likely to develop iron deficiency than those who do not (14, 15). Different types of sporting activities have been related to significant differences in iron levels which have an impact on women’s chances of developing an iron deficiency thought to be due to low dietary iron consumption and iron loss through menstruation and hormonal response to training (16, 17).

Since intensive exercise is accompanied by the formation of active forms of oxygen, Se is of particular importance to athletes. In particular, the activity of the enzyme glutathione peroxidase, a highly effective antioxidant enzyme, depends largely on the presence of Se. These Se properties can potentially be applied to improve sporting performance and recovery after training (18). The use of Se additives can increase athletic performance as well as protect muscle tissue from oxidative processes (19, 20). It is shown that correction of microelements deficiency in athletes increases their endurance and sportsmanship (21). Using an variable microelement dosing mode (e.g., oral, intravenous, transdermal) is essential in order to achieve an optimal result (22). However, the first step is to determine key levels of microelements among specific populations. To date, limited research has examined this for young wrestlers.

A recent systematic review notes that micronutrient deficiencies/imbalances can play a significant role in reducing the body’s ability to adapt to physical activity (23). Based on the described above, the purpose of this work was to study the balance of minerals of young wrestlers’ bodies.

## Methods

The study was approved by the decision of the Interuniversity Ethics Committee of the A. I. Evdokimov Moscow State University (protocol No. 01-19 of January 31, 2019). All individuals participating in the study provided written informed consent. The study was conducted in winter.

In the course of our experiment, 66 male youth wrestlers aged 18-20 years with at least 3 years of intensive experience in wrestling were examined. Of these, 21 athletes (subgroup 1) were engaged in Sambo, 25 athletes (subgroup 2) in freestyle wrestling, and 20 athletes (subgroup 3) in Greco-Roman wrestling. The main training process took place in the city of Moscow. All athletes lived and trained at the same sports bases. They received a standard diet developed in the Russian Federation for wrestlers. Food was prepared centrally at the athletes’ places of residence and/or training. The control group consisted of 92 youth cadets of a Moscow military school aged 18-20 years, with previous sports training, but not involved in wrestling. They were selected as the control group due to receiving similar physical training including combat and they also received a standard diet with food prepared at the military school.

To determine the quantitative content of microelements for study participants in the experimental and control groups the hair was cut from the occipital part of the head for the entire length in an amount of at least 0.1 g. To remove the surface contamination and to degrease the hair, the IAEA-recommended hair sample preparation method was used (24). For this purpose the hair was treated with acetone for 10-15 minutes, and then washed three times with deionized water. The hair was dried at room temperature for 10-15 minutes.

A sample of hair, weighing 0.1 g was placed in a fluoroplastic liner and 5 ml of nitric acid was added. An autoclave with the sample in the insert, was placed in a microwave oven and the sample was decomposed using the decomposition program recommended by the manufacturer of the microwave. The following heating mode was used: raising the temperature to 200 °С for 5 minutes, keeping it for 5 minutes at 200 °С, and then cooling it to 45 °С. The cooled autoclave was shaken to mix the contents and the lid was opened to balance the pressure. A qualitatively decomposed sample after distillation of nitrogen oxides was a colorless or yellowish transparent solution with no undissolved particles at the bottom or on the walls of the liner. The dissolved sample was quantitatively transferred to a test tube, diluting the sample 1000 times. Working standard solutions were prepared by diluting standard stock solutions. The proportions and concentrations of elements in standard solutions were selected in such a way that after dilution of 20–50 times the concentrations of the same order were obtained with the upper boundaries of the range of elements in the hair decomposed according to standard methods (24, 25).

The studies were carried out using ICP-MS Agilent 7900 mass spectrometer (24). Analytical signals were processed with spectrometer software, using calibration dependences calculated by the least squares method, accounting and correction of the background, and whenever necessary, taking into account the mutual influence of the measured elements. The result on the display corresponded to the arithmetic average of 3 parallel measurements of the analyzed element. The standard software package from the EXCEL and STATGRAPH statistical software package was used for data analysis to compare the levels of macro- and microelements present in wrestlers and in people who were not involved in wrestling, but who had a high level of physical activity. Use of the Kolomogorov-Smirnov test indicated the data were not normally distributed, thus nonparametric statistics were used. Results are presented as median and interquartile range. The parameters were compared based on the Kruskal-Wallis test. Spearman correlations were calculated. The comparative analysis examined five macroelements (Na, K, Mg, P, Ca) and 30 microelements. Of the trace elements analyzed, the biological role in the following 7: [As], [Rb], [Sr], [Cs], [Hg], [Tl], [Pb] has not been studied and some authors attribute them to toxic 9 (25).

## Results

As shown **Table 1**, wrestlers, in comparison with the control group, had higher levels of lithium, beryllium, boron, sodium, aluminum, calcium, potassium, vanadium, magnesium, cobalt, copper, germanium, rubidium, strontium, cadmium, antimony, caesium, barium and thallium. The most pronounced differences in levels including greater arsenic in subgroup 1, and silver in subgroup 2. All wrestlers had lower titanium and iodine than control group participants. Silicon was lowest among subgroup 3, and selenium was lowest in subgroup 2. No significant differences were found for the rest of the analyzed macro- and microelements.

**Table 1:** The content of the analyzed macro- and micro-elements in the hair (μg/g) of representatives of the experimental (n = 66) and control (n = 92) groups.

Elements	subgroup 1 median (Q1 ; Q3)	subgroup 2 median (Q1 ; Q3)	subgroup 3 median (Q1 ; Q3)	control median (Q1 ; Q3)
**lithium**	**0,20 (0,15 ; 0,22)**	**0,18 (0,08 ; 0,21)**	**0,16 (0,031;0,227)**	**0,021 (0 ; 0,065)**
**beryllium**	**0,00036 (0,00023 ; 0,00049)**	**0,00042 (0,00028 ; 0,00060)**	**0,00053 (0,00037 ; 0,00083)**	**0 (0 ; 0)**
**boron**	**1,52 (1,43 ; 1,85)**	**1,38 (1,30 ; 1,69)**	**1,71 (1,59 ; 2,29)**	**0,96 (0,58 ; 1,46)**
**sodium**	**459 (295 ; 1163)**	**446 (174 ; 589)**	**417 (214 ; 523)**	**141 (81,8 ; 253)**
magnesium	69,7 (53,5 ; 83,6)	52,8 (41,1 ; 74,9)	55,8 (44,1 ; 76,5)	70,7 (47,7 ; 101)
**aluminum**	**14,5 (11,6 ; 18,5)**	**10,3 (9,09 ; 13,1)**	**10,5 (9,19 ; 13,2)**	**9,38 (7,44 ; 11,5)**
**silicon**	**298 (289 ; 321)**	**255 (213 ; 331)**	**231(213 ; 249)**	**280 (219 ; 370)**
phosphorus	122 (116 ; 161)	118 (114 ; 169)	147 (130 ; 158)	138 (95,5 ; 159)
**potassium**	**153 (122 ; 336)**	**132 (95,7 ; 345)**	**188 (107 ; 239)**	**96,7 (54,0 ; 155)**
**calcium**	**793 (672 ;1062)**	**650 (417 ; 830)**	**586 (471 ; 838)**	**474(374 ; 657)**
**titanium**	**0,72 (0,56 ; 0,95)**	**0,73 (0,50 ; 1,04)**	**0,83 (0,61 ; 1,18)**	**1,30 (0,64 ; 3,03)**
**vanadium**	**0,022 (0,019 ; 0,029)**	**0,018 (0,013 ; 0,029)**	**0,021 (0,017 ; 0,026)**	**0,012 (0,008 ; 0,023)**
chromium	0,34 (0,30 ; 0,41)	0,32 (0,26 ; 0,49)	0,39 (0,31 ; 0,45)	0,35 (0,22 ; 0,78)
**manganese**	**0,78 (0,57 ; 0,91)**	**0,47 (0,39 ; 0,78)**	**0,38 (0,30 ; 0,70)**	**0,30 (0,19 ; 0,47)**
iron	14,4 (12,6 ; 15,7)	12,5 (9,07 ; 19,0)	13,3 (12,1 ; 16,5)	17,0 (12,5 ; 23,3)
**cobalt**	**0,062 (0,061 ; 0,067)**	**0,061 (0,036 ; 0,066)**	**0,056 (0,033 ; 0,075)**	**0,016 (0,013 ; 0,025)**
nickel	0,24 (0,19 ; 0,35)	0,24(0,19 ; 0,35)	0,42 (0,32 ; 0,53)	0,37 (0,19 ; 0,61)
**copper**	**10,6 (9,0 ; 11,1)**	**8,35 (7,95 ; 10,6)**	**10,7 (9,00 ; 11,4)**	**8,02 (6,14 ; 10,6)**
zinc	93,0 (82,6 ; 127,4)	96,7 (79,6 ;128)	117 (93,0 ; 136)	105 (83,2 ; 150)
**germanium**	**0,13 (0,12 ; 0,14)**	**0,13 (0,11 ; 0,14)**	**0,14 (0,13 ; 0,15)**	**0,089 (0,079 ; 0,110)**
**arsenic**	**0,040 (0,029 ; 0,053)**	**0,026 (0,021 ; 0,049)**	**0,025 (0,023 ; 0,031)**	**0,022 (0,0082 ; 0,037)**
**selenium**	**0,51 (0,49 ; 0,53)**	**0,49 (0,45 ; 0,52)**	**0,53 (0,49 ; 0,57)**	**0,66 (0,55 ; 0,79)**
**rubidium**	**0,15 (0,09 ; 0,24)**	**0,080 (0,065 ; 0,216)**	**0,118 (0,082 ; 0,165)**	**0,060 (0,023 ; 0,14)**
**strontium**	**2,01 (1,15 ; 2,83)**	**1,23 (0,81 ; 1,66)**	**1,38 (0,85 ; 1,98)**	**0,66 (0,38 ; 1,56)**
molibdenum	0,036 (0,031 ; 0,041)	0,030 (0,026 ; 0,042)	0,034 (0,027 ; 0,040)	0,032 (0,023 ; 0,044)
**silver**	**0,045 (0,022 ; 0,120)**	**0,054 (0,043 ; 0,130)**	**0,087 (0,061 ; 0,126)**	**0,0310 (0,0082 ; 0,0690)**
**cadmium**	**0,062 (0,014 ; 0,137)**	**0,0106 (0,0067 ; 0,0493)**	**0,0211 (0,0079 ; 0,0451)**	**0 (0 ; 0)**
tin	0,31 (0,27 ; 0,36)	0,25 (0,21 ; 0,33)	0,34 (0,27 ; 0,42)	0,36 (0,15 ; 0,61)
**antimony**	**0,057 (0,049 ; 0,117)**	**0,048 (0,030 ; 0,091)**	**0,048 (0,037 ; 0,074)**	**0,010 (0 ; 0,030)**
**iodine**	**0,042 (0,032 ; 0,054)**	**0,049 (0,034 ; 0,069)**	**0,051 (0,032 ; 0,059)**	**0,21 (0,17 ; 0,30)**
**caesium**	**0,00085 (0,00058 ; 0,00115)**	**0,00050 (0,00045 ; 0,00091)**	**0,00057 (0,00048 ; 0,00085)**	**0 (0 ; 0)**
**barium**	**1,61 (1,33 ; 2,00)**	**1,38 (1,10 ; 1,87)**	**1,60 (1,38 ; 2,11)**	**0,82 (0,36 ; 1,59)**
mercury	0,058 (0,043 ; 0,122)	0,055 (0,033 ; 0,085)	0,037 (0,026 ; 0,073)	0,054 (0,025 ;0,147)
**thallium**	**0,00048 (0,00038 ; 0,00085)**	**0,00045 (0,00022 ; 0,00078)**	**0,0006 (0,00012 ; 0,00074)**	**0 (0 ; 0)**
lead	0,89 (0,60 ; 1,60)	0,45 (0,27 ; 0,73)	0,42 (0,33 ; 0,92)	0,18 (0,12 ; 0,54)

Differences significant with p < 0.05 are shown in bold.

**Table 2** presents the significant correlation of mineral levels among the wrestlers. Strong positive correlations were observed for sodium with potassium and rubidium, magnesium with calcium, potassium with rubidium, and rubidium with caesium. All statistically significant correlations were positive.

**Table 2:** Spearman correlations of mineral content in wrestlers (N = 66).

Mineral	magnesium	potassium	calcium	titanium	vanadium	chromium	cobalt	iron	manganese	arsenic	rubidium	cadmium	tin	antimony	lead	barium	cesium
sodium	-0,06	**0,86**	-0,09	**0,55**	**0,32**	**0,53**	0,23	**0,36**	0,22	**0,48**	**0,85**	**0,43**	**0,39**	**0,55**	**0,42**	**0,51**	**0,73**
magnesium		-0,18	**0,92**	-0,15	**0,28**	-0,04	**0,40**	0,14	**0,53**	-0,07	-0,16	0,23	0,13	0,02	0,11	0,18	-0,09
potassium			-0,15	**0,65**	**0,32**	**0,62**	0,24	**0,32**	0,13	**0,49**	**0,98**	**0,51**	**0,45**	**0,54**	**0,53**	**0,56**	**0,75**
calcium				-0,14	**0,27**	-0,03	**0,39**	0,03	**0,59**	-0,12	-0,14	**0,34**	0,14	0,00	**0,24**	**0,25**	-0,07
titanium					**0,35**	**0,55**	0,06	**0,45**	0,10	**0,36**	**0,64**	**0,43**	**0,52**	**0,44**	**0,42**	**0,44**	**0,63**
vanadium						**0,58**	**0,65**	**0,61**	**0,64**	**0,40**	**0,31**	**0,56**	**0,62**	**0,53**	**0,45**	**0,36**	**0,49**
chromium							**0,45**	**0,56**	**0,35**	**0,38**	**0,61**	**0,50**	**0,60**	**0,67**	**0,48**	**0,43**	**0,56**
cobalt								**0,52**	**0,65**	0,19	0,24	**0,50**	**0,44**	**0,37**	**0,44**	**0,36**	**0,35**
iron									**0,53**	**0,38**	**0,33**	**0,37**	**0,50**	**0,58**	**0,33**	0,20	**0,45**
manganese										**0,32**	0,11	**0,52**	**0,37**	**0,48**	**0,53**	**0,35**	**0,29**
arsenic											**0,49**	**0,40**	**0,52**	**0,58**	**0,56**	**0,41**	**0,46**
rubidium												**0,51**	**0,48**	**0,57**	**0,54**	**0,56**	**0,78**
cadmium													**0,70**	**0,51**	**0,69**	**0,62**	**0,56**
tin														**0,46**	**0,62**	**0,53**	**0,52**
antimony															**0,61**	**0,38**	**0,65**
lead																**0,47**	**0,61**
barium																	**0,51**

Significant correlations with p < 0.05 are shown in bold.

## Discussion

Macronutrients such as sodium, calcium, potassium, and magnesium may increase in wrestlers’ bodies as a result of considerable sport activities and a higher content of these substances in their diets (26), for example, through consumption of sports supplements (27). Conversely, students in Polish sports schools had inadequate intake of potassium, calcium, sodium, copper, iron, zinc, phosphorus, iodine and a number of vitamins with food (28). It may be that differences in the standard diets of wrestlers and military cadets resulted in the differences found in this study for macro- and microelements.

A systematic review examined the effect of mineral content in food and supplements on sports performance. 17,433 articles were analyzed; and 130 experiments from 128 studies were included. Retrieved articles included iron (n = 29), calcium (n = 11), magnesium, (n = 22), phosphate (n = 17), zinc (n = 9), sodium (n = 15), boron (n = 4), selenium (n = 5), chromium (n = 12) and multi-mineral articles (n = 5). No relevant articles were identified for copper, manganese, iodine, nickel, fluorine or cobalt. Only iron and magnesium included articles of sufficient quality to be assigned as ‘strong’. Currently, there is little evidence to support the use of food supplementation to improve physiological markers of athletic performance with the possible exception of iron (in particular, biological situations) and magnesium as they currently have the strongest quality evidence (23).

Our results partially overlap with those described by Zaitseva and Zaitsev (29) who found an increase in the content of macronutrients Ca, Mg, P, K, Na in young wrestlers’ bodies. They believed that the increased content of macronutrients in the hair more likely indicated an enhanced “circuit” metabolism in athletes, rather than their excess (29). Indeed, a number of works describe a change in the content of mineral substances during physical exertion. For example, in adolescence, at the initial stage of sports, the development of hyperelementosis of iron, lead and selenium is noted (30). In elder men, an increase in calcium during sport activities is described (31, 32). In women, higher Ca, Cr, Fe, Co, and Zn and lower Hg are observed (31). However, in the study of cyclic sports athletes no differences in the levels of Mn, Co, Zn and Se were found, depending on the phase of the training process (32).

Many of the trace elements that were investigated in our paper have not been studied previously. Only a few studies have examined changes in the content of trace elements in sports (7, 33–35). There are no systematic reviews so far. And to date, the few available research results are contradictive. In some cases, the study was made with small groups (33). Sometimes the authors do not specify the sport for which a similar study was conducted (7). There are descriptions of a higher content of tin, rubidium and antimony in individuals involved in sports, compared with non-occupants (34). These changes are especially pronounced in individuals with an aerobic-anaerobic type of exercise compared to individuals with an aerobic type (33). Long-distance runners have an increased level of Be, Cd, Cs and Pb compared to non-active sports (7). The levels of cadmium (Cd), tungsten (W), tellurium (Te), beryllium (Be), and lead (Pb) were shown to be higher in sports (34). University students with increased physical activity had decreased hair copper, vanadium, bismuth, and mercury content in comparison to low physical activity university students (35).

Elite long distance runners have higher levels of Co, Cu, Mn, Mo, Se and Zn compared to non-athletes. During a six-month training period, these differences become more significant for Co, Cu, Mo, and Se. According to the authors, such changes reflect the process of adaptation to physical activity (33).

The above articles contradict the study of Muñoz et al. (5), who showed that the concentrations of Co and Mn are higher, and Cu is lower in individuals who are not involved in sports, compared with non-athletes (36). However, a small sample size should be noted: 26 non-athletes and 21 middle and long distance runners.

Our study found lower iodine levels in wrestlers, which requires further study of thyroid function. This is important, because we propose that low iodine levels may be associated with decreased athletic performance. For example, for high efficiency of the training process, vegan athletes need iodine (37). Lower physical fitness scores for girls have been significantly correlated with lower iodine levels, although additional research is needed (38).

The literature also describes a decrease in selenium content in runners during training (39) similar to what we found in this study. Similar changes have been described in athletes of aerobic, anaerobic and mixed sports (8, 40). A non-systematic review found that during sports the level of oxidative stress increases, which can lead to a deficiency of selenium in athletes. According to the authors, those involved in sports need additional administration of selenium with food to protect the body from possible free radical damage (41).

Wrestlers in our study were characterized by higher levels of several macro- and microelements, including more toxic ones than controls. We studied a greater number of minerals than in other similar studies, which adds to the available scientific literature for examining the relationship between levels of macro and microelements and sports training. Our findings are preliminary. The study is limited by a lack of explanation regarding why these differences existed (i.e. they could be due to nutrition; training; physiological impacts of exercise; body composition difference; physical environment; and so on). As we did not track dietary intake, we are unable to state whether differences were due to what was eaten rather than the physical training done by each group. We also did not directly track the physical training of each group. Additional research is warranted to further explore underlying factors for the differences found.

Practical recommendations: It is necessary to monitor the level of trace elements in athletes-wrestlers during their training. In particular, it may be necessary to control their intake with food and/or food additives. Restoring an optimal balance of macro- and microelements can contribute to better physical performance of wrestlers. Special attention should be paid to the function of the thyroid gland in athletes-wrestlers, because we found they had a pronounced iodine deficiency. Perhaps this category of athletes needs additional iodine supplementation and/or thyroid hormones. It is necessary to consider the issue of prescribing selenium-containing drugs to wrestlers to correct the deficiency of this trace element and prevent oxidative stress associated with high-intensity training.

## Data availability statement

The raw data supporting the conclusions of this article will be made available by the authors, without undue reservation.

## Ethics statement

The study was approved by the decision of the Interuniversity Ethics Committee of the A. I. Evdokimov Moscow State University. The patients/participants provided their written informed consent to participate in this study.

## Author contributions

Conceptualization, KG. Formal analysis, VP and AK. Funding acquisition, IK. Investigation, MY and IS. Methodology, DK and OZ. Project administration, KG and VZ. Resources, ND and EB. Supervision, MO and AS. Writing original draft, VZ and KG. Writing—review & editing, KH. All authors have read and agreed to the published version of the manuscript. All authors agree to be accountable for the content of the work.

## Acknowledgments

This paper has been supported by the RUDN University Strategic Academic Leadership Program.

## Conflict of interest

The authors declare that the research was conducted in the absence of any commercial or financial relationships that could be construed as a potential conflict of interest.

## Publisher’s note

All claims expressed in this article are solely those of the authors and do not necessarily represent those of their affiliated organizations, or those of the publisher, the editors and the reviewers. Any product that may be evaluated in this article, or claim that may be made by its manufacturer, is not guaranteed or endorsed by the publisher.
